# Getting the word out: neural correlates of enthusiastic message propagation

**DOI:** 10.3389/fnhum.2012.00313

**Published:** 2012-11-26

**Authors:** Emily B. Falk, Matthew Brook O'Donnell, Matthew D. Lieberman

**Affiliations:** ^1^Department of Communication Studies, University of MichiganAnn Arbor, MI, USA; ^2^Institute for Social Research, University of MichiganAnn Arbor, MI, USA; ^3^Department of Psychology, University of MichiganAnn Arbor, MI, USA; ^4^Department of Psychology, Psychiatry and Biobehavioral Sciences, University of CaliforniaLos Angeles, CA, USA

**Keywords:** fMRI, sentiment analysis, natural language processing, information diffusion, word-of-mouth

## Abstract

What happens in the mind of a person who first hears a potentially exciting idea?We examined the neural precursors of spreading ideas with enthusiasm, and dissected enthusiasm into component processes that can be identified through automated linguistic analysis, gestalt human ratings of combined linguistic and non-verbal cues, and points of convergence/divergence between the two. We combined tools from natural language processing (NLP) with data gathered using fMRI to link the neurocognitive mechanisms that are set in motion during initial exposure to ideas and subsequent behaviors of these message communicators outside of the scanner. Participants' neural activity was recorded as they reviewed ideas for potential television show pilots. Participants' language from video-taped interviews collected post-scan was transcribed and given to an automated linguistic sentiment analysis (SA) classifier, which returned ratings for evaluative language (evaluative vs. descriptive) and valence (positive vs. negative). Separately, human coders rated the enthusiasm with which participants transmitted each idea. More positive sentiment ratings by the automated classifier were associated with activation in neural regions including medial prefrontal cortex; MPFC, precuneus/posterior cingulate cortex; PC/PCC, and medial temporal lobe; MTL. More evaluative, positive, descriptions were associated exclusively with neural activity in temporal-parietal junction (TPJ). Finally, human ratings indicative of more enthusiastic sentiment were associated with activation across these regions (MPFC, PC/PCC, DMPFC, TPJ, and MTL) as well as in ventral striatum (VS), inferior parietal lobule and premotor cortex. Taken together, these data demonstrate novel links between neural activity during initial idea encoding and the enthusiasm with which the ideas are subsequently delivered. This research lays the groundwork to use machine learning and neuroimaging data to study word of mouth communication and the spread of ideas in both traditional and new media environments.

## Introduction

When I muse about memes, I often find myself picturing an ephemeral flickering pattern of sparks leaping from brain to brain, screaming “Me, me!”

- Douglas Hofstadter (Hofstadter, 1985)

### Message propagation

How does an idea move from being an ordinary idea to a leaping spark, potentially spreading like wildfire to an entire society? What happens in the mind of a person who first hears a *potentially* exciting idea, reads a *potentially* viral story online, or adopts a *potentially* contagious new behavior? What processes determine whether that person will go on to promote the idea, story or behavior? Message propagation from one person to the next is one of the oldest forms of advertising, and a powerful form of social influence (Subramani and Rajagopalan, 2003; Brown et al., 2007; Christakis and Fowler, 2007; Smith et al., 2007; Fowler and Christakis, 2008, 2010). Understanding the mechanisms that underlie this form of social influence is critical, especially given the rise of social media and abundance of data afforded by the new media environment.

A considerable body of literature has examined the spread of ideas from the point of view of message recipients [for a review, see (Berger, 2012)]. For example, psychologists have characterized the factors that lead message recipients to be persuaded by arguments (Petty and Cacioppo, 1986a,b; Chaiken et al., 1989; Eagly and Chaiken, 1993; Albarracin et al., 2005; Eagly and Chaiken, 2005). In parallel, communication scholars have described the ways in which information flows from sources such as the mass media to the general population (Katz and Lazarsfeld, 1955; Katz, 1957; Nisbet and Kotcher, 2009), as well as how innovations diffuse through populations (Rogers, 1995). Recent work from a marketing perspective has examined content and context-based factors that predict when online content, such as news items, are likely to go viral (Wojnicki and Godes, 2008; Berger and Milkman, 2012) and when online reviews are influential (Godes and Mayzlin, 2004; Chevalier and Mayzlin, 2006; Trusov et al., 2009; Chintagunta et al., 2010). The new media environment more broadly, and social media in particular, have also spurred renewed interest in so-called “earned” marketing (in contrast to paid ads) in which individuals promote ideas, products or behaviors enthusiastically within their social networks.

Relatively less research, however, has focused on the underlying mechanisms that precede enthusiastic message propagation from the point of view of the communicator [cf., research on motives (Dichter, 1966; Engel et al., 1993; Sundaram et al., 1998; Hennig-Thurau et al., 2004; Buechel and Berger, 2011)]. There may be comparatively less of this kind of research in the social psychological literature due to methodological challenges, including participants' lack of conscious access (Nisbett and Wilson, 1977) to the underlying factors that lead them to perform later behaviors (including enthusiastically supporting an idea) and the fact that invoking such introspection can contaminate subsequent psychological and behavioral processes (Wilson and Schooler, 1991).

### New combinations of methods for studying the mechanisms of message propagation

Although limited in its own ways (Poldrack, 2008), functional magnetic resonance imaging (fMRI) can measure neural responses in the moment that participants are initially processing messages. Functional MRI interrogates several neurocognitive networks simultaneously, without contaminating the process by explicitly demanding self-reports of the psychological processes that occur during any given task. Hence, fMRI may be a useful tool to study the processes underlying and promoting social communication. In particular, fMRI may be useful in understanding the precursors of message propagation, especially if there are processes set in motion during initial exposure to ideas that are not consciously registered by the individual taking in the information. Already, a body of research has demonstrated the successful use of fMRI to study naturalistic social communication. For example, fMRI has been used to measure the degree of synchronization between different observers being exposed to the same thirty-minute film (Hasson et al., 2004). Prior research has also demonstrated that increased synchrony between the brains of a speaker and listeners are associated with increased effectiveness of communication (Stephens et al., 2010).

In addition, tools from fields such as natural language processing (NLP) may provide insights regarding participant sentiment that are not captured using other methods. Recent studies of online social networks, such as Twitter and Facebook, have demonstrated the value of applying tools from NLP and information retrieval to richly linked and socially situated language data (O'Connor et al., 2010; Bakshy et al., 2011). Sentiment analysis (SA) of Twitter, for example, has been used discover opinions regarding new products (Go et al., 2009), identify regional dialects (Eisenstein et al., 2010) and retrospectively predict political movements (Eisenstein et al., 2010; Tumasjan et al., 2010). Sentiment analysis of news content has also been used to understand the characteristics of messages that are shared most often (Berger and Milkman, 2012). Sentiment analysis uses machine learning algorithms to train classifiers to distinguish between text samples grouped according to some attribute (e.g., positive vs. negative sentiment) on the basis of a selection of linguistic features (i.e., use of certain combinations of adjectives, nouns, 1st person pronouns, etc.) (Kim and Hovy, 2006; Pang and Lee, 2008).

In should be noted that the state-of-the-art in SA techniques are restricted primarily to language form (i.e., written text or transcripts of linguistic events) and not to features of communication such as non-verbal cues, prosody and intonation, which might intuitively seem to be strongly associated with enthusiastic message sharing. However, lexical choice and formal linguistic patterns underlie and transmit meanings and culture (Sinclair, 2004) and are linked with individuals' emotion and cognitive experience (Pennebaker, 2011). Automatic linguistic analysis is able to capture these patterns of language usage that are typically not easy for human coders to recognize spontaneously or consistently.

In parallel, expressive behavior is often represented through non-linguistic vocal cues, body language, and other features that may not be captured by the automated sentiment analyzer (SA), but which may predict important outcomes related to how successfully ideas spread. Across multiple content areas and contexts, gestalt ratings, or thin-slices, of expressive behavior, rated by human coders, have been shown to accurately predict a range of important outcomes (Ambady and Rosenthal, 1992), and neural systems associated with shared sensorimotor representation have been implicated in empathic accuracy (Zaki et al., 2009). Thus, the human coded scores provide a way of examining neural processes associated with transmission of ideas that may not be captured by automatic SA, and also provide a point of comparison for neural correlates of the automatic SA that may not be consciously registered by human coders.

In the present investigation, we combined the use of fMRI with SA and human coding to examine the underlying neural processes that precede enthusiastic message propagation behavior, and in particular, the valence and evaluative content of how messages are shared. More specifically, we interrogated the neural signals present during initial exposure to ideas, and their relationship to the ways in which the initial idea recipient subsequently transmitted the idea to others in a videotaped session following the fMRI scan. We examined the neurocognitive correlates of communicator enthusiasm as identified by the specific qualities of language employed (using automated SA), and by human gestalt impressions of the corresponding behavior.

### Neural systems associated with successful communication

In contrast to prior studies that have investigated social influence from the perspective of message recipients, in the present investigation we investigate successful social influence from the perspective of the message communicator. More specifically, we were interested in the neural processes that precede spreading ideas with enthusiasm. We suggest that three key sets of processes may support spreading ideas with enthusiasm.

First, to the extent that ideas resonate with the message communicator upon initial receipt, she/he may be better positioned to advocate those ideas to others in an enthusiastic manner. Activity in the medial prefrontal cortex (MPFC) in Brodmann's area 10 (BA10) and precuneus/ posterior cingulate cortex (PC/PCC) have been associated with self-related processing (Lieberman, 2010), as well as subsequent behavior change following exposure to persuasive messages (Falk et al., 2010, 2011; Chua et al., 2011). In addition, the neuroeconomics literature has characterized the ventromedial prefrontal cortex (VMPFC) and ventral striatum (VS) as encoding reward and value signals (Knutson et al., 2001; McClure et al., 2004; Knutson and Cooper, 2005; Haber and Knutson, 2010). These regions might be more active to the extent that participants connect with ideas initially.

Second, consideration of how ideas might be received by others is likely to play a key role in the level of enthusiasm that one expresses outwardly when describing that idea to others (Krauss and Fussell, 1991; Higgins, 1992). Neural activity in dorsomedial prefrontal cortex (DMPFC) and temporal-parietal junction (TPJ) are commonly associated with social cognition, perspective taking and mentalizing about the views of others (Lieberman, 2010; Saxe, 2010). In our prior work, individual differences in participants' abilities to persuade others of the value of their preferred ideas was associated exclusively with activity in TPJ (Falk et al., 2012b). In the current investigation, we hypothesized that TPJ and DMPFC would be associated with evaluating ideas with respect to their social value, and hence would predict the enthusiasm with which ideas were subsequently propagated. Activity in VS and VMPFC are also associated with exposure to stimuli that are popular or valued by others (Plassmann et al., 2008; Zaki et al., 2011), and with conforming to the opinion of others (Campbell-Meiklejohn et al., 2010). In the context of preparing to share ideas, beyond encoding one's own evaluation of the incoming ideas, the VS and VMPFC might also encode value with respect to ideas in the social context. Likewise, activity in MPFC increases during exposure to socially tagged stimuli, compared to stimuli where the preferences of others are unknown (Mason et al., 2009). In sum, beyond merely taking in information and evaluating one's own preferences, the neural precursors of spreading ideas with enthusiasm are likely to include the mentalizing system and other neural systems that encode the potential social value of ideas.

Third, our participants were exposed to ideas during the scanning session, and videotaped discussing ideas approximately half an hour later, following the scanning session. Thus, neural systems in the medial temporal lobe (MTC) including the hippocampus, implicated in memory encoding and retrieval (Cabeza and Nyberg, 2000), as well as PC/PCC, implicated in retrieval of autobiographical memories, may be associated with participants' ability to speak enthusiastically about the ideas when given the opportunity. To the extent that the ideas were more richly encoded, participants may have been able to draw on their memory of the idea content to provide descriptions that were later coded as more enthusiastic.

Finally, prior neuroimaging research has demonstrated that activity in each of the neural systems above is associated with increased synchrony between the brains of a speaker and a listener. More specifically, the MPFC, PC/PCC, TPJ as well as the medial temporal lobes (MTL) and striatum are associated with increased effectiveness of communication (Stephens et al., 2010).

### Summary of hypotheses

We hypothesized that the neural precursors of enthusiastic message propagation would share common neural underpinnings with several sub-processes associated with successful speaker-listener communication. More specifically, activity in regions that have been implicated in self-related processing (including MPFC, PC/PCC), reward (including VS, VMPFC), mentalizing (DMPFC, TPJ), and memory (including the MTL) during initial idea encoding may be associated with later enthusiasm expressed for those ideas. We further hypothesized that the neural patterns associated with enthusiasm as defined by automated linguistic SA, and separately by gestalt human ratings would overlap, despite relying on different pathways to capturing the underlying concept of “enthusiasm.” In particular, the gestalt ratings made by human coders should capture elements of non-verbal behavior that are not captured by the SA, whereas SA may capture patterns of social communication that are not consciously registered by human coders.

### Links to prior work on the neural correlates of successful message propagation

In prior work (Falk et al., 2012b), we examined the neural activity of participants from this same experiment, in combination with the behavioral responses of a second group of individuals who viewed video-taped interviews from the current group of participants. The goal of our prior work was to characterize the neural processes that were activated by specific ideas that were destined to spread, as well as individual differences in the tendency to be a good “idea salesperson” (someone who successfully persuades others of the value of their idiosyncratic preferences). In that investigation, we found that participants' intentions to spread ideas covaried with increased activity in MPFC and PC/PCC. By contrast, individual differences at the subject level in ability to persuade others of one's idiosyncratic preferences were reflected in one region that is often associated with social cognition and perspective taking during initial idea exposure—TPJ. Finally, ideas that spread successfully, regardless of message communicator, were associated with increased activity in a combination of these regions (PC/PCC, TPJ) as well as DMPFC and VS (Falk et al., 2012b).

In the present investigation, we examine a different set of constructs that speak to the behavior of our initial participants (those who were scanned during initial idea exposure). In particular, a considerable body of social psychological literature has demonstrated that intentions are often related to the behaviors that follow, but are not synonymous; indeed, there is often a gulf between what we intend to do, and the actual execution (Fishbein and Ajzen, 1975; Armitage and Conner, 2001; Fishbein et al., 2001; Webb and Sheeran, 2006). Whereas our prior analyses did not examine any specific features of the initial participants' communication, in the present investigation, we explored the overlap and divergence in the neural systems associated with cues identified by automated SA of the participants' descriptions and human perceived enthusiasm. Indeed, although there should be overlap in the neural correlates of participants' intentions to share ideas, and the actual enthusiasm with which they subsequently spread the ideas, these metrics are only modestly correlated.

## Materials and methods

### Participants

Twenty participants were recruited from an undergraduate subject pool and through mass emails and posted fliers, and received either course credit or financial compensation for their participation; one participant was dropped due to technical difficulties, resulting in a usable sample of nineteen participants (11 female, mean age = 20.55, SD = 6.17). All participants were right-handed, and spoke English fluently. Participants also met the following criteria related to fMRI safety: (1) were not claustrophobic; (2) had no metal in their bodies (other than tooth fillings); (3) were not pregnant/breast-feeding. Potential participants were excluded if they were currently taking psychoactive medication. Informed consent was obtained from all subjects in accordance with the policies of the UCLA Institutional Review Board.

### Procedure (Figure 1)

After arrival and consent at the fMRI center, participants were asked to pretend that they were interns at a television studio. During the primary scanning session, each participant viewed and heard 24 descriptions of television show ideas while their neural activity was recorded using fMRI. Following each description, participants rated how likely they would be to recommend the show to their producer [results related to these data reported in Falk et al. (2012b)]. After exiting the scanner, participants were video taped discussing each show, with the idea that the videotape would be shown to their producer for final decisions about which shows would be produced.

### Stimuli

**Figure 1:**
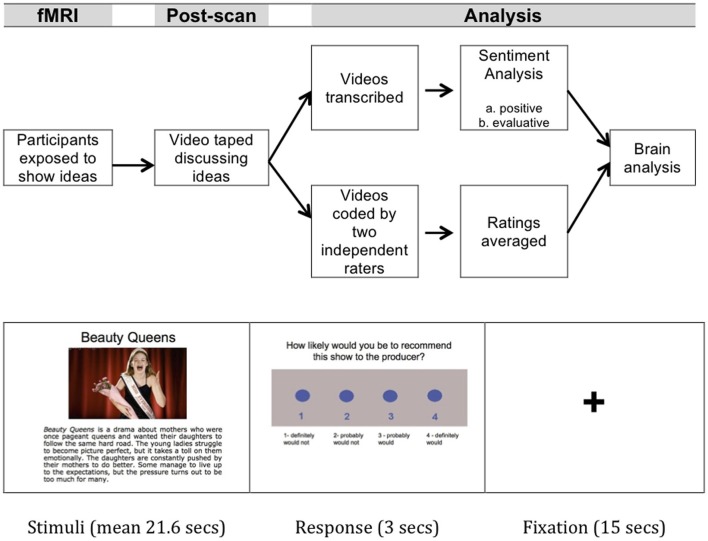
**Participant procedure and data analysis flow.** Participants were exposed to an initial set of ideas while neural activity was monitored throughout their brains using fMRI. They were then videotaped discussing each idea. These videotapes were coded by humans, and transcripts of the language were separately given to an automated language classifier that performed a sentiment analysis.

Preliminary ideas for television show pilot episodes were generated by UCLA undergraduates in response to a prompt in which they were asked to “Pretend you are pitching a new TV show idea to a network.” From this pool of show descriptions, 24 show ideas were selected as final stimuli based on further pilot testing and assessment by the research team; shows were selected to appeal to a wide range of audiences and to have comprehensible plots. The language of the pilot television show descriptions was then edited by the research team to standardize grammar, spelling, description length and language complexity across shows. The show descriptions contained relatively neutral descriptions of the pilot show ideas (mean sentiment rating [positive × evaluative] = −0.01, on a scale from −1 to 1). An image representing the show was also paired with the description (see example, Figure 1).

During the scanner session, each participant was presented with all 24 show descriptions, broken into three runs with eight block per run (mean block length = 21.6 s; SD = 1.7 s; 310 s/run totaling 465 volumes/run). Fixation rest periods between blocks served as an implicit baseline. Each block contained one show description, which consisted of the show title at the top, an image representing the show, and a brief text-based plot summary (see Figure 1). All plot summaries were read aloud by the same voice, to control for participant reading speed. Following exposure to the show description, participants were prompted to indicate their intention to pass on information about the show (see Figure 1), and were given 3 s to make the rating. Blocks were separated by 15 s rest periods in which participants were presented with a fixation cross. The order of shows was counterbalanced across runs, with each participant receiving one of three different pseudorandom orderings.

### Natural language processing

We examined features of the participants' language using machine learning techniques applied to transcripts of the videotaped post-scan session. All videotaped descriptions of the show ideas collected following fMRI scanning were transcribed by trained research assistants. These transcripts were normalized (removing punctuation, transcription symbols, etc. and transformed into lower case) and then were submitted to a SA API (http://text-processing.com/api/sentiment/) that uses classifiers trained on a database of movie reviews (Pang et al., 2002; Pang and Lee, 2004; Perkins, 2011). In the SA, the text is first analyzed for descriptive vs. evaluative language, which roughly corresponds to how opinionated the description is; the classifier was trained on a corpus of sentences labeled as either evaluative [from Rotten Tomatoes (http://www.rottentomatoes.com/), a website that provides subjective reviews of movies] or descriptive [from IMDb (http://www.imdb.com/), a film-encyclopedia website that provides plot descriptions of movies]; (Pang and Lee, 2004). This is followed by valence analysis which assesses how positive or negative the language used to describe the ideas is; this classifier is trained on a corpus of movie reviews labeled either positive or negative, based on the reviewers' star ratings.

The SA uses the Naïve Bayes classifier implementation provided in the Natural Language Toolkit (NLTK) (Bird et al., 2009) and a combination of frequent words and significant bigrams (two word sequences) as features. For example, bigrams such as “really like,” “would definitely,” “completely different,” “deals with,” and “different things” are associated with positive sentiment; whereas, bigrams such as “the worst,” “interesting but,” “be ok,” “guess I,” and “boring and” are associated with negative sentiment. More descriptive text (closer to IMDB) tended to describe the content of the shows without offering a specific recommendation or opinion, whereas more evaluative content (closer to Rotten Tomatoes) tended to offer more explicit evaluations and personal opinions of the shows (see Table 1 for example text and corresponding ratings).

**Table 1:** **Example normalized text (case and punctuation removed) with associated ratings by humans who had access to full voice and visual cues (scale = 0–100), automated positivity scores from the language classifier (scale = −1 to 1), and automated evaluative language scores from the language classifier (scale = 0–1)**.

	**Higher positivity/Lower negativity**	**Lower positivity/Higher negativity**
Higher evaluative/Lower descriptive	Beautyqueens	Beautyqueens
	Beauty queens i thought looked pretty hilarious um it was about moms who were former beauty queens who raised their daughters to be beauty queens it was about the stress of um their the daughters trying to be beauty queens and it was also about the mothers a little too um and that actually looked really funny um so i would definitely recommend moving forward with that one	Beauty queens i don't know if its i am biased cause i am a guy but beauty queens would really not appeal to me that much cause the mere fact that i don't want to see a little girl putting hair and make up on for four hours go walking across the stage getting off the stage and doing it again two or three times and then losing or winning i really don't care um so that really doesn't appeal to me or jujust the moms pushing the kids jus they do that anyway why beauty
	human: 85	human: 20.5
	positivity: 0.49	positivity: −0.80
	evaluative: 0.99	evaluative: 0.99
	Bizarreworld	Bizarreworld
	Bizzare world is a proposal in which contestants would um would travel to a different country in every episode and they would have to survive in awkward in a bizarre different part of a country and it would just follow them in their show to survive and maintain in a very different environment i thought it was very interesting show because i think it's an interesting proposal because it would um attract a lot of audiences who are interested in um in knowing what there is different countries of the world and it's really interesting to see how people manage to survive so i would definitely recommend it	Um bizarre world uh i mean i have seen multiple shows like they and they wouldn't really appeal to me just you put uh people from different parts of the world into one location or dif different countries or something every week and then there is a challenge the one who performs the worst in that challenge is the one that's eliminated i mean just i i it wouldn't really appeal to me uh the one appealing factor would be that you would get to see different cultures from around the world the challenges and hohow thethey have to think on the spot
	human: 72	human: 33.5
	positivity: 0.42	positivity: −0.75
	evaluative: 0.95	evaluative: 0.99
	Nightlife	Nightlife
	Nightlife Night life is about um students i think living in other countries and they had to kind of just go through the trial of living in another country not knowing the language having to make money and support themselves um i think that would be an entertaining show um kind of like real world but in other countries so it would be entertaining i think people would really like to watch it especially it it’s just to see scenery in other countries cuz that’s always cool to see if you’ve never been there	Night life is a television show based on basically a party scene hence the title night life uh where they throw different people into different areas of the world and … and um they don't know the language they don't know the culture they don't have much money they have to get jobs um that would be an ok show i think it would make it although it would probably be a show that i would not watch
	human: 77	human: 35
	positivity: 0.4182	positivity: −0.7862
	evaluative: 0.9989	evaluative: 0.7409
Lower evaluative/Higher descriptive	Beautyqueens	Bizarreworld
	The next show is called beauty queens and this has to do with a bunch of moms that were beauty queens when they were young and it focuses on their daughters and how their daughters struggle with living up to their mothers expectations and like becoming beauty queens themselves and so some can live up to their mom's expectations and become beauty queens but some fail and it deals with the drama of this	hhh um the next one is called bizarre world and it's a um it's a reality show where they take starting with like ten contestants they take them to um to all these different locales where they have to um undergo challenges based on the culture and the environment they that they're in and that i guess who ever uh which ever contestant doesn't uh does the worst with these challenges is uh is eliminated its its kind of standard reality show fair
	human: 39.5	human: 36.5
	positivity: 0.41	positivity: −0.56
	evaluative: 0.01	evaluative: 0.41
	BizarreWorld	Roommates
	Um oh the next one is geared towards a more reality tv show called bizarre world and this is where there's a big group of contestants and each episode they are taken to a different new and bizarre place around the world and they are forced with challenges that have to do with the environment that they're placed in and obviously the ones who don't cope with the challenges are kicked off the show and those that um cope well continue until they're down to the final winner kind of like this show survivor now which is really popular so this could be popular as well	The next one is called roommates and it's um four girls that are randomly placed together and they go to college and become roommates and they each have a distinct personality it's kind of like the previous show called classes but geared to a more older group of watchers
	human: 78	human: 76.5
	positivity: 0.45	positivity: −0.56
	evaluative: 0.21	evaluative: 0.04
Example where automated text rating is similar, but human coded scores differ, likely due to non-verbal cues	Nightlife	NightLife
	um: night life was a show about the five people in the different country in the foreign country and sort of how they have to survive um: and make their own living: and deal with the changes in a different country	the next show is called night life and has to do with teenagers in foreign countries and they have to learn about the culture and um kind of their interactions and their night life
	human: 64.5	human: 40
	positivity: 0.48	positivity: 0.47
	evaluative: 0.00	evaluative: 0.00

Human, human coded enthusiasm scores; positivity, automated sentiment analyzer rating of positivity (vs. negativity); evaluative, automated sentiment analyzer rating of evaluative (vs. objectivity).

The SA's evaluative vs. descriptive ratings were transformed to range from 0 (completely neutral/objective/descriptive) to 1 (completely polar/subjective/evaluative) (variable called evaluative). The SA's positivity vs. negativity ratings were transformed to range from −1 (most negative) to 1 (most positive) (variable called positive). From these scores, we also computed a combined score in which valence ratings were weighted by the degree to which the language employed was evaluative (positive × evaluative). Examples of text that were rated by the classifier as high and low along each dimension are presented in Table 1.

### Video coding

Separately, human coders viewed the videotaped recordings of participants transmitting ideas following the scanning session and made ratings of the participants' gestalt enthusiasm for each idea. Two trained research assistants (one male, one female) watched each video and made an assessment of how enthusiastic the participant was about each idea using a feeling thermometer (0 = very cold/completely unenthusiastic–100 = very warm/completely enthusiastic). Coders were instructed to form a gestalt impression of the speaker's enthusiasm for the show. There was a high degree of correspondence between the assessments made by the two raters (*r* = 0.932). The two raters' assessments were thus averaged together to form the human-coded enthusiasm rating. Example text and ratings for human-coded enthusiasm scores are also presented in Table 1.

In sum, our analysis process produced four assessments related to the way in which ideas encoded during the scanning session were subsequently expressed: human gestalt coding of each participants' enthusiasm for each idea, machine classified sentiment valence (how positive vs. negative was the language used in each participants' description of each idea?), machine classified evaluative vs. descriptive language (how descriptive versus evaluative was each participants' description of each show idea?), and a combined score in which sentiment valence was weighted by the degree to which evaluative (vs. descriptive) language was employed; this index captures stronger positive recommendations of the ideas (referred to as positive × evaluative). Each of these assessment types was correlated with neural activity during the initial encoding of ideas in order to identify neural precursors of each effect (details in section “Statistical Analysis”). In addition, we examined the overlap between the neural correlates of enthusiasm as captured by the different dimensions of the automated SA, and as captured by the human coders, and points of divergence.

### fMRI data acquisition

Imaging data were acquired using a Trio 3 Tesla head-only MRI scanner at the UCLA Ahmanson-Lovelace Brainmapping Center. Head motion was minimized using foam padding and surgical tape; goggles were also fixed in place using surgical tape connecting to the head coil and scanner bed. A set of high-resolution structural T2-weighted echo-planar images were acquired coplanar with the functional scans (spin-echo; TR = 5000 ms; TE = 34 ms; matrix size = 128 × 128; 33 interleaved slices; FOV = 220 mm; slice thickness = 4 mm; voxel size = 1.7 × 1.7 × 4.0 mm; flip angle = 90°). A high resolution T1-weighted magnetization prepared rapid acquisition gradient echo (MP-RAGE) scan was also acquired (TR = 2300 ms; TE = 2.47 ms; matrix size = 64 × 64; FOV = 256 mm; slice thickness = 1.0 mm; 160 slices; voxel size = 1.3 × 1.3 × 1.0 mm; flip angle = 8°). Three functional runs were recorded for each participant (echo-planar T2-weighted gradient-echo, TR = 2000 ms, TE = 30 ms, flip angle = 75°, matrix size = 64 × 64, 33 axial slices, FOV = 220 mm, 4 mm thick; voxel size = 3.4 × 3.4 × 4.0 mm). Each run consisted of eight blocks (one show was described and rated in each block). Each run lasted for 310 s, totaling 465 volumes. The first two volumes from each run were discarded to allow the scanner to equilibrate.

### Statistical analysis

#### fMRI preprocessing

Functional images were despiked using the default options in AFNI 3dDespike, and all data were visually inspected to ensure completeness. All subsequent preprocessing was carried out using Statistical Parametric Mapping (SPM8, Wellcome Department of Cognitive Neurology, Institute of Neurology, London, UK). In SPM8, functional images were corrected for slice acquisition timing differences within volumes (slice order interleaved), realigned within and between runs to correct for residual head motion, and coregistered using a two stage process in which the mean functional volume was coregistered with the matched-bandwidth structural scan, and the matched-bandwidth structural scan was coregistered with the MPRAGE, using 6-parameter rigid body transformations. Segmentation was applied to ensure accurate skull stripping. The coregistered, segmented MP-RAGE scans were then normalized into Montreal Neurological Institute (MNI) standard stereotactic space (based on the MNI152_T1_1 mm template) and the resulting parameters were applied to all segmented, coregistered, functional images. The resulting images were spatially smoothed using a Gaussian kernel (8 mm full width at half maximum).

#### Individual level effects

We examined the neural processes present during idea encoding that were associated with the subsequent way in which the ideas were conveyed, separately for each participant. Design matrices were created for each participant in SPM8, modeling activity that was greater during exposure to the show descriptions in the scanner, than during rest, and correlating this task-related activity with parametric modulators based on each of the constructs of interest. Task-related activity during exposure to show ideas was modeled as a boxcar from onset of the voiced reading of the issue until offset (mean description length = 21.6 s; SD = 1.7). Parametric modulators of this boxcar function were derived from the human-coded enthusiasm scores of each idea, and then separately, each of the dimensions produced by the automated language classifier (evaluative, positive sentiment, positive sentiment weighted by evaluative). The stimulus rating period was modeled as a covariate of no interest. Parametric modulation analysis allowed us to compare not just on-off (e.g., subject liked vs. disliked the show), but instead, to model task related neural activity in relation to the subsequent enthusiasm with which the idea was described (as rated by human coders, or using key outputs of the SA). In other words, a series of first level models were computed in which enthusiasm (and other relevant dimensions produced by the SA) were separately regressed onto task-related brain activity in order to identify neural regions associated with each psychological process, for each participant. We also ran a set of additional analyses in which pairs of variables (e.g., human-coded enthusiasm + positive sentiment coded by classifier) were entered as predictors in a multiple regression framework at the single subject level in SPM in order to identify shared variance between constructs.

#### Group level effects

For each of the four parametric modulation analyses conducted at the single subject level, a group level random-effects model was constructed, averaging across participants. The group level models employed one-sample *t*-tests in order to average across beta contrast values computed from first level models. These maps contain information about common precursors of each post-scan construct of interest (e.g., enthusiasm expressed about each show, post-scan). In addition, conjunction analyses were conducted in order to examine the neural overlap in regions associated with human coded enthusiasm, and the components identified by the automated language classifier. Results for all primary effects of interest are reported at a threshold of *p* < 0.005, with a *k* = 37 voxel extent, controlling the rate of false discoveries at *p* < 0.05 based on a Monte Carlo Simulation implemented using AlphaSim in the software package AFNI (Ward, 2000) using a whole-brain mask. Conjunction analyses were conducted using this same threshold for each component effect (*p* < 0.005, *k* = 37, corresponding to *p* < 0.05 corrected). All coordinates are reported in MNI space.

## Results

### Show ratings

Transcripts of participants discussing show ideas were coded using an automated NLP classifier, trained on a corpus of film reviews (Pang et al., 2002; Pang and Lee, 2004; Perkins, 2011), along dimensions of sentiment valence (positive vs. negative feelings about the shows) and the degree to which the language used was evaluative (vs. purely descriptive). We also computed a combined score in which sentiment valence was weighted by how evaluative the language was for each participant, for each show, in order to capture stronger endorsements. Videotapes of each participant discussing each show idea were rated for gestalt enthusiasm by independent human coders.

Participants varied substantially in the show ideas that they found most compelling (this was true along all coded dimensions); intraclass correlation coefficients (ICCs) grouping by subject and by show are presented in Table 2. The ICCs suggest that most of the variance in show ratings occurred within subjects and within shows (individual subjects were not systematically more or less enthusiastic about shows in general; there was not a systematically high degree of enthusiasm for one set of shows and a systematically low degree of enthusiasm for other shows). However, the ICC magnitudes suggest that there was more between-subject variability in the ratings produced by the automated language classifier than in the ratings produced by the human coders. Human coded enthusiasm was weakly, positively associated with both the automated classifier's positive valence ratings (average correlation across individuals: *r* = 0.29) as well as with evaluative language (average correlation across individuals: *r* = 0.21), but not with the interaction of valence and evaluative language (average correlation across individuals: *r* = 0.003). Examples of text and corresponding ratings are presented in Table 1.

**Table 2:** **Intraclass correlation coefficients representing the proportion of variance in each rating of interest that occurred between subjects (or between shows), compared to the total (between + within) variance**.

**Variable**	**ICC (Grouping**	**ICC (Grouping**
	**by subjects)**	**by shows)**
Human coders	0.02	0.14
Automated positivity	0.18	0.13
Automated evaluative	0.23	0.18
Positivity scaled by evaluative	0.05	0.09

ICCs were calculated as [Tau / (Tau + SigmaSq)] using the mult.icc function from the multilevel package (Bliese, 2008) in R (R Development Core Team, 2011).

### Neural precursors of positive sentiment (classified by NLP)

**Figure 2:**
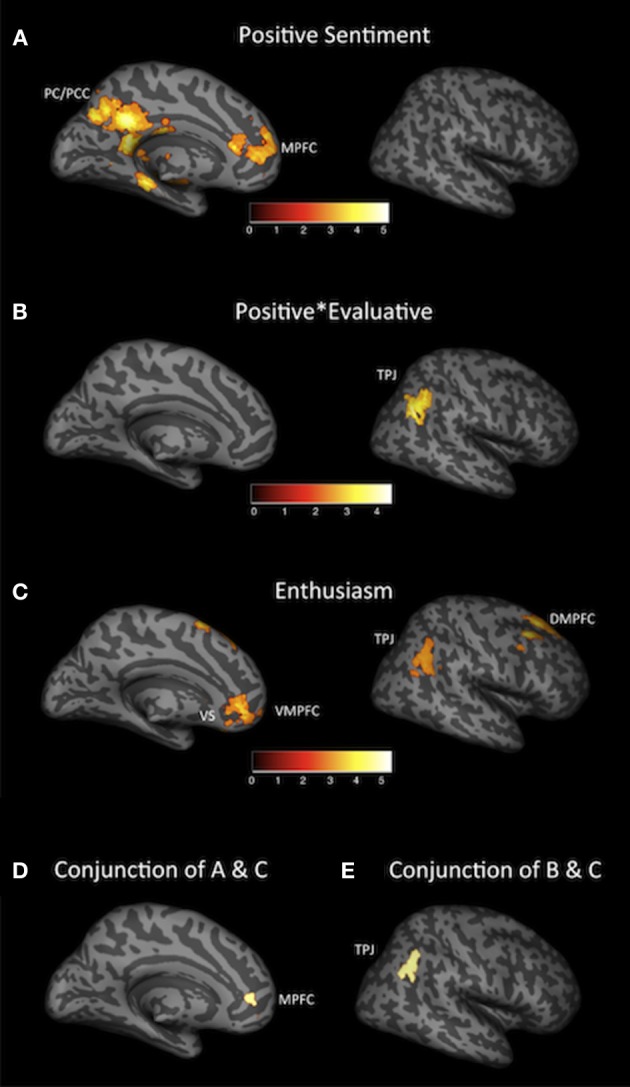
**Neural regions associated with the ways in which ideas were communicated after the scan. (A)** Associations with positive sentiment, as rated by the automated sentiment analyzer. **(B)** Associations with positive sentiment, scaled by evaluative, as rated by the automated sentiment analyzer. **(C)** Associations with enthusiasm, as rated by human coders. **(D)** Conjunction analysis of **(A)** (associations with positive sentiment, as rated by the automated sentiment analyzer) and **(C)** (associations with enthusiasm, as rated by human coders). **(E)** Conjunction analysis of **(B)** (associations with positive sentiment, scaled by evaluative, as rated by the automated sentiment analyzer) and **(C)** (associations with enthusiasm, as rated by human coders). Note: All analyses were conducted using a threshold of *p* < 0.005. Results in **(A–C)** employ a cluster extent threshold of *k* = 37, corresponding to *p* < 0.05, corrected for multiple comparisons. Results in **(D)** and **(E)** represent conjunctions of analyses cluster corrected in this manner. DMPFC, dorsomedial prefrontal cortex; MPFC, medial prefrontal cortex; PC/PCC, precuneus/posterior cingulate; TPJ, temporal parietal junction; VMPFC, ventral medial prefrontal cortex; VS, ventral striatum.

More positive sentiment toward the shows, as rated by the automated classifier, was associated with increased activity at the time of first exposure to show descriptions in MPFC and PC/PCC, as well as activity in DMPFC, dorsal striatum and MTL (Figure 2A and Table 3A). More evaluative positive sentiments (statements that were more opinionated in their positivity, i.e., closer to positive ratings provided on rotten tomatoes than in IMDB, captured by the sentiment valence × evaluative metric) were exclusively associated with increased activity in TPJ (Figure 2B and Table 3B). No regions were significantly associated with the evaluative dimension on its own, or with more negative sentiment.

**Table 3:** **Associations between neural activity in participants' brains and ratings of interest of their post-scan descriptions**.

**Table 3A:** **Positive correlations with positive sentiment, as rated by the automated sentiment analyzer**.

**Region**	**Local max**			***K***	***t*-stat**
	***x***	***y***	***z***		
Precuneus	−6	−67	37	594	3.97
Posterior cingulate	−6	−47	31	–	5.12
L cerebellum	−6	−47	−2	–	5.62
MPFC	−2	56	10	208	4.02
MPFC	−6	50	1	–	3.61
ACC	1	32	16	–	4.11
MTL	−20	−30	−11	108	4.26
MTL	−13	−5	−26	–	3.75
DMPFC	−13	43	34	51	3.82
R Cuneus	11	−84	19	45	3.63
R cerebellum	46	−54	−29	50	4.21
Middle frontal gyrus	−26	67	22	45	3.9

**Table 3B:** **Positive correlations with positive sentiment, scaled by evaluative, as rated by the automated sentiment analyzer**.

**Region**	**Local max**			***K***	***t*-stat**
	***x***	***y***	***z***		
TPJ	49	−60	40	78	3.68
–	42	−71	37	–	4.61

### Neural precursors of expressing enthusiasm (rated by human coders)

Exposure to show ideas that were subsequently described more enthusiastically (as rated by human coders) was associated with increased neural activity in regions previously associated with memory encoding and retrieval (MTL), self-related processing (MPFC and PC/PCC), reward and the computation of value (VMPFC and VS), social cognition and mentalizing (DMPFC and TPJ) and the mirror neuron system [inferior parietal lobe (IPL), dorsal premotor cortex (dPMC)] (Figure 2C and Table 3C).

**Table 3C:** **Positive correlations with enthusiasm, as rated by human coders**.

**Region**	**Local max**			***K***	***t*-stat**
	***x***	***y***	***z***		
MPFC	1	56	7	188	3.44
VMPFC	−5	50	−11	–	3.02
Subgenual ACC	4	29	−8	–	4.42
TPJ	49	−57	28	66	3.22
IPL/Angular gyrus	42	−57	34	–	3.24
TPJ/Angular gyrus	−47	−64	31	99	2.97
Superior parietal lobule/IPL	−30	−71	46	–	4.43
DMPFC	11	39	43	194	3.5
dPMC	35	22	61	–	5.43
DMPFC	−12	42	37	260	2.99
dPMC	−16	32	58	–	5.31
MTL/Parahippocampal gyrus	18	−23	−20	64	5.25

### Conjunctions of NLP-coded sentiment and human coded enthusiasm

A conjunction analysis indicates that activity in MPFC and DMPFC was associated both with positively valenced sentiment, as coded by the automated classifier, and the enthusiasm ratings coded by humans (Figure 2D and Table 3D). A separate conjunction analysis suggests that the activity in TPJ, which was the sole neural region associated with the sentiment scores weighted by the degree to which the language used was evaluative was also associated with human-coded enthusiasm ratings (Figure 2D). Although our results were discussed in terms of our *a priori* regions of interest (with full lists of activations given in the tables), it should be noted that these MPFC, DMPFC, and TPJ conjunction effects represent the only significant regions in the brain—not just in our *a priori* regions of interest.

**Table 3D:** **Conjunction analysis of (a) positive correlations with positive sentiment, as rated by the automated sentiment analyzer and (c) positive correlations with enthusiasm, as rated by human coders**.

**Region**	**Local max**			***K***
	***x***	***y***	***z***	
DMPFC	−13	43	37	12
MPFC	−2	53	4	33

### Distinctive contributions of human-coded enthusiasm and SA

Finally, in order to verify the overlap between these effects across analyses, we ran a set of additional analyses in which pairs of variables (e.g., human-coded enthusiasm + positive sentiment coded by classifier) were entered as predictors in a multiple regression framework at the single subject level in SPM. Human coded enthusiasm and results from the SA explain overlapping variance in each of the neural regions that are the focus of this investigation, with a few notable exceptions. Human coded enthusiasm, controlling for scores generated by SA was associated with activity in dPMC, IPL and MTL (Tables 3F, G). In other words, human coded enthusiasm scores accounted for variance in these regions that was not captured by SA (Figure 2E and Table 3E). No regions remained significantly associated with either SA positivity scores or SA positive × evaluative scores, after controlling for human coded enthusiasm.

**Table 3E:** **Conjunction analysis of (b) positive correlations with positive × evaluative, as rated by the automated sentiment analyzer and (c) positive correlations with enthusiasm, as rated by human coders**.

**Region**	**Local max**			***K***
	***x***	***y***	***z***	
TPJ	45	−57	37	28

**Table 3F:** **Human-coded enthusiasm, controlling SA rated positive × evaluative**.

**Region**	**Local max**			***K***	***t*-stat**
	***x***	***y***	***z***	
MTL	−26	−43	−8	38	4.13
IPL	−30	−70	46	41	3.15
dPMC	−9	18	64	73	3.59

**Table 3G:** **Human-coded enthusiasm, controlling SA rated positive**.

**Region**	**Local max**			***K***	***t*-stat**
	***x***	***y***	***z***	
dPMC	−23	22	52	178	4.37
dPMC	35	22	61	63	4.36
IPL/superior parietal lobe	−26	−74	52	64	3.65
Parahippocampal gyrus	−16	−9	−32	74	3.99
Middle cingulate	8	1	28	41	3.89

Note: Results thresholded at p < 0.005, (whole brain: k = 37), corresponding to p < 0.05, corrected for multiple comparisons. ACC, anterior cingulate cortex; DMPFC, dorsomedial prefrontal cortex; MPFC, medial prefrontal cortex; MTL, medial temporal lobe; TPJ, temporal parietal junction; VMPFC, ventral medial prefrontal cortex; IPL, intraparietal lobule; dMPC, dorsal premotor cortex. Dashed lines (–) indicate continuation of table entry from above.

## Discussion

### Neural precursors of the spread of ideas

In the present study, we investigated the neural precursors of spreading ideas with enthusiasm from the perspective of the *message communicator*. Although a growing body of work has examined the neurocognitive underpinnings of attitude change and behavior change from the perspective of the message recipient (Klucharev et al., 2008, 2009, 2011; Mason et al., 2009; Berns et al., 2010; Campbell-Meiklejohn et al., 2010; Zaki et al., 2011), we know substantially less about what prompts people to share ideas enthusiastically. This type of investigation is especially relevant in the context of the new media environment, which facilitates word-of-mouth transmission of ideas to much wider networks.

In the current investigation, we examined the neural precursors of spreading ideas with enthusiasm as one way of beginning to understand the underlying processes that may lead to the successful spread of ideas. In particular, we dissect enthusiasm into component processes that can be uniquely identified through automated linguistic SA, through gestalt human ratings of combined linguistic and non-verbal cues, and points of convergence/divergence between the two. Given the growing desire and ability to leverage linguistic data to predict relevant outcomes (e.g., virality) in the context of the new media environment, understanding the overlap and divergence between mental processes captured by NLP and by gestalt human impressions is also of importance.

### Hypotheses

We hypothesized that the process of encoding information in a way that later results in enthusiastic dissemination might share common neural underpinnings with successful speaker-listener communication in general, and that ratings captured by an automated SA would be related to subsets of neural activity associated with gestalt human-coded ratings of speaker enthusiasm.

More specifically, we hypothesized that activity in regions that have been implicated in self-related processing (including MPFC, PC/PCC), reward (including VS, VMPFC), mentalizing (DMPFC, TPJ), and memory (including the MTL) during initial idea encoding may be associated with later enthusiasm expressed for those ideas.

More specifically, we hypothesized that ideas that resonate with a listener might also be more likely to be propagated by that individual. MPFC in BA10 and PC/PCC has been associated with self-related processing (Lieberman, 2010), as well as subsequent behavior change following exposure to persuasive messages (Falk et al., 2010, 2011; Chua et al., 2011). We hypothesized that activity in these systems would be associated with later enthusiasm in communicating ideas. Indeed, both human coded enthusiasm and SA ratings of positivity were associated with activity in these regions during initial encoding.

However, we also hypothesized that personal connection to an idea should not be sufficient to prompt enthusiastic message propagation. Instead, outward expressions of enthusiasm for ideas also require an understanding of what others are likely to value (Krauss and Fussell, 1991; Higgins, 1992), and may involve consideration of what others are likely to think of us if we share (Engel et al., 1993; Sundaram et al., 1998; Hennig-Thurau et al., 2004). Neural activity in DMPFC and TPJ are commonly associated with social cognition, perspective taking and mentalizing about the views of others (Lieberman, 2010; Saxe, 2010). We hypothesized that activity in these regions during initial encoding would be associated with participants' evaluations of ideas with respect to the value others would place on those ideas. In addition, in orthogonal analyses that we performed with this dataset, individual differences in participants' abilities to persuade others of the value of their preferred ideas was associated exclusively with activity in TPJ (Falk et al., 2012b).

Indeed, human-coded enthusiasm scores were associated with activity in both of these regions, and more positive, evaluative sentiments (as coded by SA) was associated exclusively with increased activity in TPJ. Consistent with our initial hypotheses, it is possible that during initial idea exposure, increased perspective taking could have positioned participants to later argue more enthusiastically for the merit of the ideas in describing them to others, preparation that was evident both through automated linguistic analysis and human-coding. In other words, these data are consistent with the idea that preferences and recommendations may involve a contextualization of one's own thoughts with respect to those of the group. In reflecting on social influence, Allport (1954) observed that not only are we swayed by those with whom we have direct interactions, but that we also behave in accordance with others who are “imagined or implied.” In the current investigation, we suggest the complement of this idea: that in taking in information, we may process both the value of the idea to ourselves, but also the value it is likely to have to others. To the extent that we deem the idea valuable to others, we may be more prepared to make a stronger recommendation and to argue in an evaluative fashion when describing the idea to others.

We also hypothesized that lower-level reward mechanisms could facilitate the spread of ideas from one person to the next; in this context, imagining that one will be able to tell another about a cool new television show might involve anticipation of a positive response from the other person (Engel et al., 1993; Sundaram et al., 1998; Hennig-Thurau et al., 2004). The VMPFC and VS are regions commonly associated with encoding reward and value signals (Knutson et al., 2001; McClure et al., 2004; Knutson and Cooper, 2005; Haber and Knutson, 2010). Activity in VS and VMPFC are also associated with exposure to stimuli that are popular or valued by others (Plassmann et al., 2008; Zaki et al., 2011), and with conforming to the opinion of others (Campbell-Meiklejohn et al., 2010).

Consistent with this hypothesis, we found that human-coded enthusiasm was associated with activity in VS and VMPFC during initial idea encoding. Automated SA ratings were not. One possibility is that activity in these regions tracks enthusiasm for ideas, which is subsequently encoded by non-verbal signals, or by language cues not identified with the current classification dimensions. On a broader scale, neural activity in VS and VMPFC has been shown to predict the cultural popularity of songs (Berns and Moore, 2012). In other words, beyond encoding one's own evaluation of the incoming ideas, the VS and VMPFC might also encode value with respect to ideas in the social context. Thus, although our data also cannot speak to the flow of ideas across populations, they do hint at the possibility of common neural mechanisms supporting the spread of ideas from person to person, and the ultimate popularity of those ideas in larger groups of people.

Finally, we hypothesized that memory encoding processes, which are commonly associated with neural activity in the MTL including the hippocampus, (Cabeza and Nyberg, 2000), as well as PC/PCC, implicated in retrieval of autobiographical memories, might be associated with enthusiastic message propagation. We found that both human coded enthusiasm and positivity as captured through automated SA were associated with neural activity in MTL.

In sum, we found that increased neural activity in several hypothesized networks previously associated with better speaker-listener communication (Stephens et al., 2010) are also associated with encoding of ideas that are subsequently described with enthusiasm by a message *communicator*. These regions include the MPFC, PC/PCC, VMPFC, VS, DMPFC, TPJ, and MTL. All of these regions were associated with gestalt ratings of enthusiasm as coded by trained human coders. One subset of these regions (MPFC, PC/PCC, DMPFC, and MTL) was associated with positive valence as classified through linguistic SA, whereas a different region TPJ was associated with more evaluative, positive descriptions, as coded by the SA.

In addition to activity in our hypothesized regions that have previously been implicated in social and affective processing, human-coded enthusiasm ratings were associated with neural activity in regions that are associated with shared sensorimotor-representations within the mirror-neuron system including the IPL and dPMC (Spunt and Lieberman, 2012). Indeed, these regions were the primary regions associated with human coded enthusiasm scores when controlling for automated SA ratings. Thus, although prior work in the mirror neuron system literature has primarily focused on mirroring others who are seen, this type of mental simulation may also prepare individuals to *share* ideas enthusiastically with others at later points in time.

### Connections to the default mode network

It is also of interest that the regions observed to predict transmission of ideas with enthusiasm (VMPFC, MPFC, DMPFC, TPJ, and MTL) are often characterized as *the default mode network (DMN)*. The DMN has been implicated in studies of mind wandering and other forms of stimulus independent thought; furthermore, increased DMN activity is often associated with performance decrements on tasks requiring attention or other forms of executive control (Schooler et al., 2011). The ubiquity of mind wandering has led researchers to investigate the function and benefits of this process, however, most studies highlight increased error rates and decreased task performance with increased DMN activity; few studies have explicitly demonstrated increased task performance associated with DMN activity [c.f. recent work (Meyer et al., 2012) demonstrating that increased activity in regions of the DMN are associated with better social working memory]. The current data suggest that activity in several regions of the DMN during the encoding of ideas is associated with more effective performance later on when pitching ideas to others. In other words, DMN activity is not always indicative of poor subsequent outcomes, and in fact, may be predictive of better task outcomes, when the task in question involves self-reference and/or social judgment. Regions of the DMN are also predictive of better outcomes when the “task” involves exposure to messages designed to facilitate positive behavior change (Falk et al., 2010, 2011, 2012a; Chua et al., 2011).

### Combination of neuroimaging with tools from natural language processing

Methodologically, we view this as a demonstration of the synergy of automated language analysis with neuroimaging data from fMRI studies. Although humans do not typically report specific linguistic features as contributing to their impressions of ideas, these features nonetheless are detectable by automated SA, and are associated with many of the same neural precursors as human gestalt ratings. In addition, some neural precursors are associated with SA ratings, but not human's explicit coding of communications. As such, SA, and other tools from NLP can facilitate more sophisticated understanding of the brain bases of social interaction and social cognition more broadly. For example, these tools provide a framework for analyzing data in which subjects engage in tasks that involve exposure to ideas, objects, or other socially relevant stimuli, and then provide free-form post-scan language samples expressing preferences or opinions (as opposed to relying exclusively on closed-ended reports). These methods would also allow new ways of integrating fMRI data with language recorded during other experimentally relevant social interactions (alone or in more complex groups) before, during or after the scan. The natural language data in question could include videotaped interviews (as in the present study), or other data relevant to social interaction [e.g., through the Electronically Activated Recorder; EAR; (Mehl et al., 2001), or sharing of content online (Berger and Milkman, 2012)]. The resulting language corpus can then be analyzed using NLP tools to provide metrics for sentiment, use of descriptive or interactive language features, and so on, that can be applied as parameters in the analysis of the fMRI data.

New, mobile media expand the circle of friends and acquaintances with whom individuals are in perpetual contact, and looking to for advice; these new media also create an unprecedented written record of the ways in which social influence unfolds. Combination of neuroimaging and NLP methods may also help to prospectively predict who is likely to share what, and in what manner (Eisenstein et al., 2010; Tumasjan et al., 2010; Falk et al., 2011), as well as population level behaviors (Berns and Moore, 2012; Falk et al., 2012a) which lead messages to go viral. Future work in which participants type or otherwise communicate between scanners may also be of interest.

### Future directions

Given the multiple functions of each of the regions observed, further study will be required to test the psychological relationships posited; our discussion of possible psychological interpretations of these activations should be understood as one of many possible explanations. At a broader level, however, our results suggest that there are neural signals present during the initial encoding of an idea that are associated with the subsequent way in which the idea is conveyed to others. In prior work (Falk et al., 2012b), many of the regions associated with our trained coders' enthusiasm ratings were also associated with ideas that spread successfully, though there were also points of divergence. In the present investigation, we begin to delve more deeply into the component processes that are associated with the intermediate step between wanting to share a great idea and the idea spreading through a culture. In particular, the results of our automated SA help dissect and contextualize the ratings made by human coders who assessed the enthusiasm expressed by each of the participants about each show using a gestalt heuristic. In particular, human coders' enthusiasm ratings captured an integrated picture of several systems at work during initial idea encoding.

Future research that takes the current findings as starting points for constructing a priori defined regions of interest (ROIs) could interrogate the psychology of the mental processes captured within these neural regions (e.g., by using activity within these regions to predict memory for specific ideas, perceived self-relevance of the ideas, associations with implicit attitudes, etc.). Within such a brain-as-predictor framework (Berkman and Falk, in press), activity from a priori defined ROIs could also be leveraged to forecast the likely enthusiasm of communicators in spreading ideas, or even the ultimate virality of those ideas across populations.

## Conclusion

In sum, these data provide novel evidence linking neural activity during initial idea encoding to the enthusiasm with which the ideas are subsequently delivered to others and also demonstrate the novel use of sophisticated machine learning tools to link natural language data to neuroimaging data. These results and methodology also lay the foundation to link basic neurocognitive signals collected using fMRI to complex social interactions collected outside of the scanner (e.g., recorded conversation, expression of preferences or opinions in more open ended formats), as well as to data collected through online social media (e.g., Facebook, Twitter). The explosion of new communication technologies, combined with novel analysis tools, stands to expand our understanding of how ideas spread and elucidate fundamental building blocks of social communication and culture.

### Conflict of interest statement

The authors declare that the research was conducted in the absence of any commercial or financial relationships that could be construed as a potential conflict of interest.
